# Association of burnout with depression in pharmacists: A network analysis

**DOI:** 10.3389/fpsyt.2023.1145606

**Published:** 2023-03-23

**Authors:** Mu He, Kuiliang Li, Xuejiao Tan, Lei Zhang, Chang Su, Keyong Luo, Xi Luo, Chang Liu, Mengxue Zhao, Xiaoqing Zhan, Qian Wang, Jing Cen, Jun Lv, Bangbi Weng, Zhengzhi Feng, Lei Ren, Guoyu Yang, Feifei Wang

**Affiliations:** ^1^Chongqing Medical and Pharmaceutical College, Chongqing, China; ^2^Department of Medical Psychology, Army Medical University, Chongqing, China; ^3^Department of Medical English, College of Basic Medical Sciences, Army Medical University, Chongqing, China; ^4^School of Educational Science, Chongqing Normal University, Chongqing, China; ^5^Department of Psychiatry, The 980th Hospital of PLA Joint Logistics Support Force, Shijiazhuang, China; ^6^BrainPark, Turner Institute for Brain and Mental Health and School of Psychological Sciences, Monash University, Clayton, VIC, Australia; ^7^Department of Pharmacy, The Southwest Hospital of Army Medical University, Chongqing, China; ^8^Department of Psychology, Fourth Military Medical University, Xi’an, China; ^9^Department of Developmental Psychology for Armyman, Army Medical University, Chongqing, China

**Keywords:** network analysis, comorbidity, depression, job burnout, pharmacists

## Abstract

**Background:**

Burnout and depression have overlapping symptoms, but the extent of overlap remains unclear, and the complex relationship between burnout and depression in pharmacists is rarely explored.

**Methods:**

We investigated burnout and depression in 1,322 frontline pharmacists, and explored the complex relationship between burnout and depression in those pharmacists using network analysis.

**Results:**

Network analysis showed that there were 5 communities. A partial overlap was found between burnout and depressive symptoms in pharmacists. The nodes MBI-6 (I have become more callous toward work since I took this job), D18 (My life is meaningless), and D10 (I get tired for no reason) had the highest expected influence value. D1 (I feel down-hearted and blue) and D14 (I have no hope for the future) were bridge symptoms connected with emotional exhaustion and reduced professional efficacy, respectively.

**Conclusion:**

A partial overlap exists between burnout and depressive symptoms in pharmacists, mainly in the connection between the emotional exhaustion and reduced professional efficacy and the depressive symptoms. Potential core targets identified in this study may inform future prevention and intervention.

## 1. Introduction

Numerous new drugs are emerging with the progress of science and technology, such as the artificial intelligence (1), mathematical modeling (2), and digital transformation (3) in drug production. A constant stream of new drugs increases pharmacists’ workload and raises the probability of drug use errors invisibly. Medication errors, which may occur at any stage of the medication process (4), remain the leading cause of harm to inpatients (5). Pharmacists can acquire relevant drug management skills through systematic training to effectively reduce the impact of medication errors on patients (6, 7). They bear high responsibilities as their expertise level directly affects the medical outcomes and safety of patients (8), and high career development pressures and high-intensity work environments (9, 10). Therefore, pharmacists work under great stress (11, 12), which affects their mental health, thus resulting in burnout and depression (13).

Burnout, although has no universally accepted definition (14), is a syndrome comprising emotional exhaustion, depersonalization and reduced personal accomplishment (15), and is considered as an “occupational phenomenon” (16). A high rate of burnout among pharmacists may affect the efficiency of their work and increase the risk of medication errors. The risk of burnout of pharmacists is almost comparable to that of doctors and nurses (17, 18), but has received less attention. Previous studies have shown that the rate ranges from 53 to 61% among pharmacists in the health system (19) and reaches 74.9% among community pharmacists (20). In China, pharmacists may face greater occupational stress due to the reform of the medical and health care system (21). Studies have shown that 44.31–96.2% of Chinese pharmacists had a certain degree of emotional exhaustion, 38.92–99.2% moderate to high depersonalization, and 67.07–99.4% low personal accomplishment (21, 22). In addition, burnout of Chinese pharmacists was affected by factors such as length of service, education level, professional title, monthly income and shift system (23, 24). Generally, the occupational burnout of pharmacists is associated with their mental health status (25), attitudes and behaviors toward pharmaceutical service (25), and intention to resign (26). Although not yet proven, the burnout among pharmacists is mainly associated with increased medical costs for patients, growing medical error rates, prolonged recovery time for patients, and decreased patient satisfaction scores and overall quality of care (27), which further leads to decreased work efficiency, increased absence, and raised turnover rate (26). Burnout may also increase the risk of relationship breakdown, drug abuse, depression and suicide in pharmacists (27–29).

Burnout often co-occurs with depression. They have similar symptoms and phenotypes (e.g., low energy), but are conceptually different (e.g., burnout, not depression, is specifically related to working conditions and environment) (30). A meta-analysis study showed that more than half of employees with burnout have depression (31). In the healthcare system, 16.5% of psychiatric residents in Saudi Arabia reported symptoms of burnout and depression, and a significant correlation between them was observed (32); 98% of psychiatrists in North America with moderate or severe depression have a high rate of burnout (33). Among pharmacists, the high rate of burnout is also associated with severe depression [approximately 33% (34)], but they seldom take non-pharmacological interventions (vacations, leisure, and psychotherapy) for adjustment (34). These data suggest that high co-occurrence exists between burnout and depression (35, 36), but how their symptoms are associated remains unclear.

Taken together, although the relationship between the depression and burnout has been extensively studied at the disorder/syndrome level, there is still a lack of more fine-grained symptom-level research. To solve this problem, network analysis can be used as a novel approach to explore the complex symptom-level relationship between mental disorders (37). Several recent studies have explored the symptom-level associations between symptoms of burnout and depression using network analysis. For example, network analysis among medical students showed that at least one dimension of burnout was associated with 11 of 16 symptoms (depression and anxiety symptoms), and that suicidal ideation was not associated with emotional exhaustion or depersonalization (38). Network analysis among educational staff showed that suicidal ideation was mainly associated with other symptoms of depression, but not those of burnout (39). However, it remains uncertain to which extent these results can be generalized to pharmacists. Therefore, the current study aimed to explore the complex relationship between symptoms of burnout and depression in pharmacists using network analysis.

## 2. Materials and methods

### 2.1. Ethics statement

This study was reviewed and approved by Army Medical University. Participants provided the informed consent before participating in this study. They were informed that the survey was anonymous, and was assured that personal information would not be disclosed.

### 2.2. Participants

Data were collected from November 8 to 1 December 2018 from hospitals in Chongqing, China. Hospitals in all districts and counties of Chongqing were included in the survey by stratified sampling. Data were obtained through the online questionnaire platform^1^ by forwarding the questionnaire QR code to pharmacists *via* the department head. A total of 1,663 subjects participated in the survey. The inclusion criteria included: (1) being frontline pharmacy employees or clinical pharmacists; (2) volunteering to participate in this study. The exclusion criteria included: (1) non-regular employees including interns; (2) retired pharmacists who are still working. The current study used an online survey, in which all questions were set with mandatory options. Therefore, there were no missing data. Subjects were excluded if they took fewer than 600 s or more than 1 h to complete the questionnaire, based on the length of the questionnaires. Finally, 341 participants were excluded and 1,322 were included in the final analysis, and the validity rate was 79.49% (1,322/1,663). All participants comply with the Labor Law of the People’s Republic of China, which specifies that employees should be older than 18 years, and retire at the age of 65 (male) or 60 (female). Therefore, the age of the participants ranged from 18 to 65 years.

### 2.3. Measures

#### 2.3.1. Burnout

The Maslach Burnout Inventory-General Survey (MBI-GS) is the most authoritative measure of burnout which is widely used (40). In the present study, the Chinese version of MBI-GS (41) was adopted to investigate burnout among pharmacists. The scale contains three subscales with 15 items: emotional exhaustion (5 items), depersonalization (4 items), and reduced professional efficacy (6 items). All items were scored on a 7-point frequency rating scale ranging from “0” (never) to “6” (always). A higher score indicates stronger burnout. In this study, burnout was measured as previously described by Maslach and Leiter. An emotional exhaustion score higher than 15 points indicates severe burnout, 11–15 points moderate burnout and lower than 11 points mild burnout; a depersonalization score higher than 12 points indicates severe burnout, 8–12 points moderate burnout and lower than 8 points mild burnout; a reduced professional efficacy score higher than 22 points indicates severe burnout, 18–22 points moderate burnout and lower than 18 points mild burnout. The Cronbach’ α of the total scale is 0.89, and the Cronbach’ α of emotional exhaustion, depersonalization and reduced professional efficacy is 0.93, 0.90, and 0.87, respectively.

#### 2.3.2. Depressive symptoms

The Chinese version of the Self-Rating Depression Scale (SDS) (42), one of the most widely used tools for investigating depressive symptoms (43), was used to measure the depression level among pharmacists. SDS includes 20 items involving emotional and physical symptoms, among which 10 items represent negative experiences, such as “I feel depressed and gloomy,” and 10 represent positive experiences and are scored in reverse, such as “I eat as much as before.” A standard SDS score of 50 (original score, 40) indicates clinically significant symptoms of the following three severity levels: a standard score of 25–49 (original score, 20–40) is classified as normal; 50–59 (original score, 41–47) as mild to moderate; 60–69 (original score, 48–55) as moderate to severe; and 70 and above (original score, 56 and above) as severe (44). In the current study, the Cronbach’ α for SDS was 0.88.

### 2.4. Data analysis

Data were sorted with Excel software, and data analysis was performed using R software (version 4.0.3) and its software package. Before network analysis, we normalized all skewed distribution data using non-paranormal transformation as previously described (45, 46). The goldbrick function in the *networktools* package was utilized to identify redundant nodes in the network (threshold = 0.25, *p* = 0.05). No redundancy was identified between SDS and MBI-GS nodes, but there were 4 pairs of redundant nodes within the scale, such as MBI-1 and BMI-4. We decided not to further delete any of these nodes considering the content of the nodes and the structure of the scale (47).

In order to explore the potential pathway between symptoms of burnout and depression, we assessed the network structure of depression and burnout among pharmacists *via* the Gaussian graphical model (GGM). The network was assessed using the *EBICglasso* function of the R-package *qgraph* (48). A graphical Least Absolute Shrinkage and Selection Operator (GLASSO) was used to reduce the weak edges and turn small edges into zero-weight edges to create a sparse network structure since the weak connections may make the network complex and redundant. The estimated networks were undirected. The variables in the network are “nodes” and the connections between the nodes are “edges” which is usually known as regularized partial correlation. In the network structure, the red edge indicates a negative correlation, and the blue edge a positive correlation. A thicker edge indicates a stronger correlation and vice versa.

The Walktrap algorithms in the *igraph* package were utilized to detect communities of nodes in the network. Walktrap algorithm, which is a hierarchical clustering algorithm based on the principle that short random walks tend to stay in the same community. Starting from a totally non-clustered partition, the distance between all adjacent nodes was calculated. Then, two adjacent communities were chosen and merged into a new one and the distance between communities was updated (47). The R-package *qgraph* was used to calculate the centrality index and visualize the network structure (49), and the *bootnet* package was used to test the node centrality difference (50). We found negative edges in the network of burnout and depression, in which expected influence showed greater stability than other indicators of centrality (51). The expected influence centrality is the sum of edge weights (e.g., correlation coefficient) connected by each node, suggesting that the activation of a certain symptom may activate other symptoms (52). In addition, the predictability of each node was estimated using the R-package *mgm* (53). Predictability is defined as the variance in a node which is explained by all its neighboring nodes.

Bridge symptoms are defined as the overlapping symptoms of two mental disorders (54). In this study, R-package was used to identify the nodes where depression overlaps with burnout (55). As the best index to identify bridge nodes, the centrality of bridge expected influence was calculated. Bridge symptoms indicate a greater risk of contagion to other communities, and eliminating bridge symptoms can prevent the spread of one disease to another (55). All nodes in this study were categorized into two groups: one contained twenty depressive symptoms, and the other contained fifteen burnout nodes.

The accuracy and stability of the network were evaluated using the *bootnet* function of R-package (50). First, the bootstrap analysis was performed to assess the accuracy of edge weights by calculating the 95% confidence interval (CI). A narrower CI indicates a more accurate assessment of edge weights. Then, the case-dropping bootstrap was run to examine the stability of the centrality index by calculating the correlation-stability (CS) coefficient (nboot = 2,000). The CS coefficient can rule out cases with the largest percentage when the correlation between the original and the subsample centrality indexes remains at a minimum of 0.70 (95% probability). A previous study suggested that the CS should not be lower than 0.25, but be preferably higher than 0.50 (50).

The network comparison test (NCT) function (56) in *networktools* package was used to compare the network structures and overall connectivity of five subgroups: gender, age (youth group: 18–30 years old and middle-aged and elderly group: 30–65 years old), education level (graduate group and undergraduate group), professional title (intermediate and senior professional title group and junior professional title group), and length of service (within 10 years group and more than 10 years group). The subgrouping was performed based on our research interests and the sample size of each group. The global strength value and *p*-value of each subgroup network were calculated to reflect the significance of structure invariances between two networks. Bonferroni-Holm (BH) method was used for correction of each network edge (57, 58). Network structure indicates the maximum difference of edges between networks; network edge invariance represents the difference in weight of a single edge between networks; and global strength means the sum of the weighted absolute values of all edges in each network (56).

## 3. Results

### 3.1. Descriptive statistics

Demographic information, burnout and depression levels are presented in Table 1. A total of 1,322 participants were included in the analysis, among whom 375 were male, accounting for 28.37%. Most participants (92.89%) were younger than 50 years old. The majority (64.30%) had bachelor’s degrees, and only a few had doctor’s degrees. Nearly 80% of the participants had more than 5 years of work experience. Overall, 40.62% of the participants reported moderate/high levels of emotional exhaustion; 25.34% moderate/high depersonalization and 23.30% moderate/high reduced professional efficacy, and 11.95% moderate and higher levels of clinically relevant depression, with a mean standard score of 47.35 ± 12.10. A significant association was found between the three dimensions of burnout and depression (Supplementary Table 1).

**TABLE 1:** The general situation of the participants (*N* = 1,332).

Variables	*N* (%)	*M* (SD)
**Gender**		
Male	375 (28.37)	
Female	947 (71.63)	
**Age (years)**		
18–30	449 (33.96)	
30–40	565 (42.74)	
41–50	214 (16.19)	
51–65	94 (7.11)	
**Diploma**		
Doctor	12 (0.91)	
Master	206 (15.58)	
Undergraduate	850 (64.30)	
Junior college	254 (19.21)	
**Working times (years)**		
< 5	360 (19.67)	
5–10	412 (31.16)	
11–20	284 (21.48)	
> 20	266 (20.12)	
**Burnout**		
Emotional exhaustion (score > 10)	537 (40.62)	10.50 (6.27)
Depersonalization (score > 7)	335 (25.34)	11.43 (4.68)
Reduce professional efficacy (score > 17)	308 (23.30)	9.59 (8.26)
**Depression**		
Clinically relevant symptoms of depression (Standard score > 62)	158 (11.95)	
Prevalence of depression (Standard score > 52)	433 (32.51)	

### 3.2. Network structure

**FIGURE 1:**
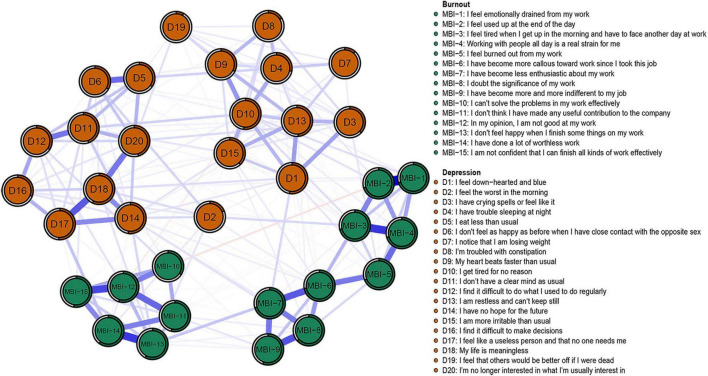
Network structure of burnout and depression symptoms in pharmacists.

The descriptive analysis of the burnout and depressive symptoms among pharmacists is presented in Table 2, and the network structure is shown in Figure 1. The network was sparse, in which 233 of the 595 potentially connected edges had connections, and the average predictability of all nodes was 0.47. The edges with the strongest connection were those of MBI-1 (I feel emotionally drained from my work) and MBI-2 (I feel used up at the end of the day) (weight = 0.45), MBI-3 (I feel tired when I get up in the morning and have to start another day at work) and MBI-4 (Working with people all day is a real strain for me) (weight = 0.35), MBI-13 (I don’t feel happy when I finish some things on my work) and MBI-14 (I have done a lot of worthless work) (weight = 0.35), and MBI-12 (In my opinion, I am not good at my work) and MBI-15 (I am not confident that I can finish all kinds of work effectively) (weight = 0.32). The edges D17 (I find it difficult to do what I used to do regularly) and MBI-14 (weight = 0.10), D14 (I have no hope for the future) and MBI-13 (weight = 0.07), D1 (I feel down-hearted and blue), and MBI-7 (I have become less enthusiastic about my work) (weight = 0.07) had strongest connection between depression and burnout. There was a relatively narrow bootstrapped 95% CI, which implies that certainty and accuracy may exist in the edges of the burnout-depression network (Supplementary Figure 1). Difference between edge weights by the bootstrapped difference test is shown in Supplementary Figure 2.

**TABLE 2:** Abbreviation, mean, standard deviation, expected influence, and predictability of symptoms in SDS and MBI-GS.

	Abbreviation	Mean (SD)	EI	Predictability
I feel emotionally drained from my work	MBI-1	2.32 (1.38)	0.42	0.700
I feel used up at the end of the day	MBI-2	2.55 (1.49)	0.02	0.701
I feel tired when I get up in the morning and have to face another day at work	MBI-3	2.11 (1.47)	0.96	0.710
Working with people all day is a real strain for me	MBI-4	2.14 (1.46)	0.65	0.706
I feel burned out from my work	MBI-5	1.37 (1.35)	0.25	0.688
I have become more callous toward work since I took this job	MBI-6	1.31 (1.32)	1.28	0.721
I have become less enthusiastic about my work	MBI-7	1.26 (1.29)	0.89	0.703
I doubt the significance of my work	MBI-8	1.23 (1.39)	0.43	0.632
I have become more and more indifferent to my job	MBI-9	1.15 (1.33)	−0.46	0.554
I can’t solve the problems in my work effectively	MBI-10	1.59 (1.72)	−1.40	0.342
I don’t think I have made any useful contribution to the company	MBI-11	1.68 (1.90)	0.10	0.538
In my opinion, I am not good at my work	MBI-12	1.28 (1.68)	0.46	0.547
I don’t feel happy when I finish some things on my work	MBI-13	1.63 (1.81)	0.78	0.577
I have done a lot of worthless work	MBI-14	2.11 (1.88)	0.64	0.601
I am not confident that I can finish all kinds of work effectively	MBI-15	1.28 (1.58)	0.19	0.526
I feel down-hearted and blue	D1	1.47 (0.75)	0.39	0.529
I feel the worst in the morning	D2	2.69 (1.00)	−1.97	0.190
I have crying spells or feel like it	D3	1.19 (0.50)	−0.70	0.370
I have trouble sleeping at night	D4	1.74 (0.94)	−0.60	0.278
I eat less than usual	D5	2.06 (1.02)	−0.09	0.344
I don’t feel as happy as before when I have close contact with the opposite sex	D6	2.52 (1.08)	−0.34	0.333
I notice that I am losing weight	D7	1.30 (0.69))	−2.31	0.126
I’m troubled with constipation	D8	1.47 (0.84)	−1.63	0.169
My heart beats faster than usual	D9	1.38 (0.68)	−0.48	0.324
I get tired for no reason	D10	1.73(0.88)	1.30	0.520
I don’t have a clear mind as usual	D11	2.19 (0.93)	0.77	0.449
I find it difficult to do what I used to do regularly	D12	2.17 (0.98)	−0.50	0.320
I am restless and can‘t keep still	D13	1.40 (0.70)	0.82	0.487
I have no hope for the future	D14	2.24 (1.00)	1.03	0.523
I am more irritable than usual	D15	1.61 (0.83)	−0.05	0.379
I find it difficult to make decisions	D16	2.80 (0.92)	−1.07	0.311
I feel like a useless person and that no one needs me	D17	2.25 (0.97)	0.53	0.512
My life is meaningless	D18	2.34 (0.98))	1.31	0.571
I feel that others would be better off if I were dead	D19	1.22 (0.58)	−2.30	0.082
I’m no longer interested in what I’m usually interest in	D20	2.10 (0.96)	0.69	0.455

SD, standard deviation; EI, expected influence; SDS, Self-Rating Depression Scale; MBI-GS, Maslach Burnout Inventory-General Survey.

**FIGURE 2:**
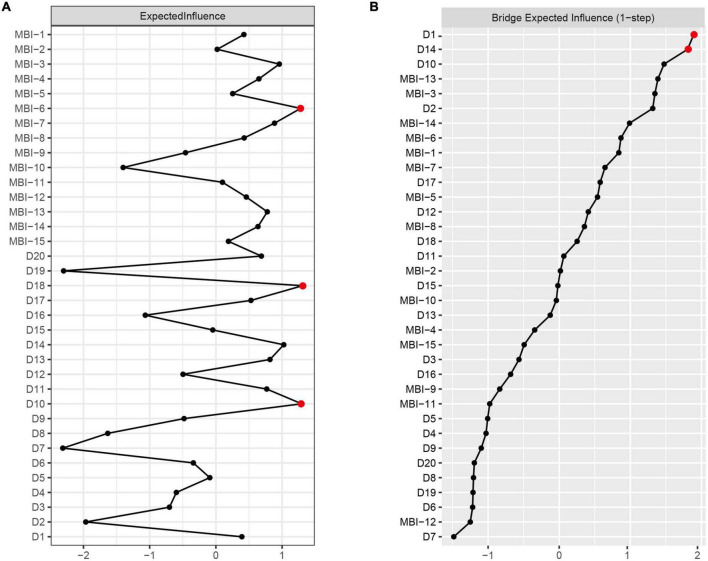
Centrality plot of the expected influence **(A)** and bridge expected influence **(B)** of burnout and depression symptoms in the network (z-score).

Expected influence centrality is shown in Figure 2A. MBI-6 (I have become more callous toward work since I took this job), D18 (My life is meaningless), and D10 (I get tired for no reason) had the highest expected influence, indicating that they were core nodes in the network. To ensure the stability of these expected influence centrality estimates, the CS coefficient of node expected influence (value = 0.75) was > 0.5, demonstrating that the centrality index was fairly stable (Supplementary Figure 3). The difference between node expected influence was shown by bootstrapped difference test (Supplementary Figure 4). The most central nodes (i.e., MBI-6, D18, and D10) had significantly higher expected influence estimates than less central nodes.

### 3.3. Bridge nodes

The centrality of bridge expected influence is shown in Figure 2B. The nodes D1 (I feel down-hearted and blue) and D14 (I have no hope for the future) had the highest bridge expected influence. The CS coefficient of bridge expected influence (value = 0.75) was > 0.5, demonstrating that the bridge centrality index was fairly stable (Supplementary Figure 5). Supplementary Figure 6 showed the difference between bridge expected influence by bootstrapped difference test, indicating that D1 and D14 had a significantly higher bridge expected value than the remaining variables.

### 3.4. Community analysis

Five clusters of burnout and depression network were determined with Walktrap algorithms, three of which constituted MBI-GS, which is consistent with the three dimensions reported by Maslach and Leiter (59): MBI-1-5 for emotional exhaustion, MBI-6-9 for depersonalization, and MBI-10-15 for reduced professional efficacy. The remaining two clusters were negative depression description (D1/D3/D4/D7/D8/D9/D10/D13/D15/D19) and positive depression description (D2/D5/D6/D11/D12/D14/D16/D17/D18/D20).

### 3.5. Network comparison test analysis

The network structures of the five subgroups are presented in Supplementary Figure 7. Differences in the network structures of burnout and depressive symptoms were observed between pharmacists with length of service, but no significant difference was found in global strength [Network variance (M) = 0.19, *p* = 0.04; Global strength variance (S) = 0.50, short length of service group: 15.23, long length of service group: 15.73, *p* = 0.15], indicating that there were differences in interactions between nodes but no difference in overall connectivity. No difference was found in other four subgroups: gender subgroup [Network variance (M) = 0.15, *p* = 0.57; Global strength variance (S) = 0.33, short length of service group: 15.37, long length of service group: 15.70, *p* = 0.51], age subgroup [Network variance (M) = 0.14, *p* = 0.70; Global strength variance (S) = 0.07, youth group: 15.56, middle-aged and elderly group: 15.49, *p* = 0.86], education level subgroup [Network variance (M) = 0.18, *p* = 0.43; Global strength variance (S) = 0.62, postgraduate group: 14.98, undergraduate group: 15.59, *p* = 0.50], and professional title subgroup [Network variance (M) = 0.14, *p* = 0.49; Global strength variance (S) = 0.14, senior professional title group: 15.55, junior professional title group: 15.41, *p* = 0.69].

## 4. Discussion

This study explored the complex relationship between burnout and depression among pharmacists using network analysis. The standardized score of the SDS scale was equivalent to that of Chinese nurses (48.5 to 52 points), but slightly higher than that of doctors (43.45 to 47.4 points) (60–62). The prevalence of moderate and high levels of clinically relevant depressive symptoms was 11.95%, which was similar to the prevalence (15.7%) reported by French pharmacists before the breakout of COVID-19 (34), but significantly lower than the prevalence (44.8%) reported by community pharmacists in Qatar during COVID-19 pandemic (29). Despite the fact that they adopted different measurement tools, they are still comparable in terms of clinical criteria. Participants also reported a high level of burnout, with 40.62% reporting a moderate/high level of emotional exhaustion, 25.34% depersonalization, and 23.30% reduced professional efficacy. This is lower than the proportion of pharmacists who reported burnout during the epidemic, with 53.1%, 50.8%, and 69.5% reporting a moderate/high level of emotional exhaustion, depersonalization, and reduced professional efficacy, respectively (29). The three dimensions of burnout were significantly associated with depression, consistent with the findings of a previous systematic review (31).

Despite the significant association between burnout and depression, network connectivity showed that the burnout and depression network was relatively sparse, with 233 of 595 potentially connected edges (39.16%) having distinct connections. This was similar to the network connectivity of burnout, depression and anxiety among medical students in previous studies, with 34.4% of edges having non-zero connections (38). However, the connection rate was lower than that of previous studies using either MBI-GS (47) or Zung SDS (63). These pieces of evidence suggest that burnout and depression may be correlated only at some nodes. The analysis of the network structure and community showed that the emotional exhaustion and reduced professional efficacy of burnout were more closely related to two communities of depression, and were more likely to have overlapping symptoms, while depersonalization was weakly associated with depression. In addition, the suicide-related node D19 was weakly associated with the three dimensions of burnout, consistent with previous reports about medical students and educational personnel (38, 39). Data suggest that burnout and depression are closely related, but each has a specific component (64). Our findings provide an explanation for the controversy on the overlap between depression and burnout (38).

The centrality of network show that the nodes with the highest expected influence value are D18 (My life is meaningless), D10 (I get tired for no reason), and MBI-6 (I have become more callous toward work since I took this job), indicating that these nodes may have the greatest impact on the whole network structure and may be considered as potential targets for related intervention. The main responsibilities of clinical pharmacists in China include the routine work such as communicating with newly admitted patients, participating in ward rounds, discussing medication plans, monitoring medication, providing consultation guidance, delivering pharmaceutical lectures and teaching, as well as some other challenging and innovative work (65). A study on Vancouver General Hospital and Richmond Hospital revealed that clinical activities accounted for 82% of pharmacists’ work time, while non-clinical activities only 18%, of which relaxation accounted for 2%, and educational lectures 2% (66). This finding suggests that attention should be paid to the scheduling of pharmacists’ work time to balance between work and rest *via* increasing proper leisure activities. The burnout node MBI-6 connected emotional exhaustion and depersonalization with two clusters of depression. Nodes D18 and D10 were the two communities of depression, which connected the depressive symptoms and burnout nodes of their communities (59). These three nodes connect to a maximum of other nodes in each community, which may affect the entire network. For example, maintaining a work-life balance, finding joy in life and enriching oneself can be beneficial in reducing mental health problems and burnout among medical staff (67–69). Therefore, the intervention aimed at the above nodes may indirectly affect the activation and maintenance of other nodes, thereby affecting the entire network and reducing the burnout and depressive symptoms of pharmacists.

The results of bridge centrality show that the nodes with the highest bridge expected influence are D1 (I feel down-hearted and blue) and D14 (I have no hope for the future), indicating that D1 and D14 are more strongly associated with the burnout nodes than the other nodes of depression. Therefore, intervention for D1 and D14 can effectively reduce the transmission of depressive symptoms and burnout nodes based on network analysis (55). Our results suggest that D1 is most likely the overlapping symptom between depression and emotional exhaustion, consistent with the previous finding that most people with burnout feel sad (70, 71). Pharmacists in China take responsibility for a large amount of tedious daily work such as reviewing medication lists (72). Negative emotions, such as D1, may affect their work efficiency (73–75), and lead to medication errors and other medical incidents. Unfortunately, pharmacists may encounter more death-related scenarios as medical staff, which could further increase their feelings of sadness, especially during the COVID-19 pandemic (76, 77).

In addition, D14 is most likely the overlapping symptom between depression and reduced professional efficacy, in line with the despair and helplessness found in individuals with depression and burnout (71, 78, 79). This may be related to the career planning and development of pharmacists, especially to the obstacles in their career development prospects, professional title promotion and salary (80–82). In China, special attention should be paid to pharmacists of traditional Chinese medicine. Previous studies have reported that there are low proportion of intermediate and senior professional titles, low education levels, and low rates of further education among these pharmacists (83), which may lead them to feel confused and desperate about their future. In addition, network comparison analysis pointed out the difference in the connection between length of service and symptoms. Previous studies have shown that pharmacists with more than 10 years of service scored higher in emotional exhaustion and cognitive weariness (84). Therefore, relevant management sectors should pay attention to the daily emotion of pharmacists and the burnout of senior employees, and conduct regular screening and popularization of mental health for pharmacists. Meanwhile, importance should be attached to the career development prospects of junior and senior pharmacists and improving the supporting measures to stimulate the work enthusiasm of pharmacists. Our results further confirm the complex overlap between depressive symptoms and different dimensions of burnout, which is beneficial to the understanding and differential diagnosis of the relationship between depression and burnout in pharmacists.

Several limitations in our study should be addressed. First, previous studies have reported an increase in the prevalence of depression and burnout during COVID-19; the current findings based on data collected in 2018, therefore, may not be applicable for pharmacists during the pandemic. In the future, it is necessary to further explore the relationship between depression and burnout of pharmacists during COVID-19. Second, the cross-sectional survey could not be used for causal explanation, and the results were easily influenced by the state of the subjects, which could not reflect the dynamic changes of depression. Third, on the one hand, further studies are needed to confirm whether the results can be generalized to other employees in the health system; on the other hand, samples in this study were all from Chongqing municipality in western China, so the results may not represent the situation in more developed cities considering the uneven regional economic development in China. In addition, the proposed intervention targets were not validated in this study, which requires further studies to verify the effectiveness of these intervention targets. Finally, the node redundancy may impact the network structure (85), and should be taken into consideration when interpreting current results.

## 5. Conclusion

Pharmacists play an important role in the health system. However, burnout and depression in pharmacists has received little attention. The present study used network analysis to explore the complex relationship between burnout and depression in pharmacists, and identified 5 communities. The nodes D18 (My life is meaningless), D10 (I get tired for no reason), and MBI-6 (I have become more callous toward work since I took this job) have the highest expected influence value. There is a partial overlap between burnout and depressive symptoms in pharmacists. D1 (I feel down-hearted and blue) and D14 (I have no hope for the future) are bridge symptoms connected to emotional exhaustion and reduced professional efficacy, respectively. The death-related node (D19) is weakly associated with the three dimensions of burnout. These findings increase the understanding of the overlap between burnout and depression, and provide potential targets for the prevention and intervention for burnout and depression in pharmacists.

## Data availability statement

The raw data supporting the conclusions of this article will be made available by the authors, without undue reservation. Request to access this dataset should be directed to KL, risyaiee@msn.cn.

## Ethics statement

The studies involving human participants were reviewed and approved by the Army Medical University. The patients/participants provided their written informed consent to participate in this study.

## Author contributions

QW, JC, JL, and BW: data acquisition. MH, KLi, and LR: formal analysis. MH, KLi, XT, LR, LZ, CL, XL, ZF, and FW: writing. KLi, LR, XT, LZ, CS, KLuo, MH, MZ, XL, ZF, GY, and FW: review and editing. All authors contributed to the article and approved the submitted version.

## Footnotes

1
www.wjx.cn
