# Supplementary material for: Association of burnout with depression in pharmacists: A network analysis
Source: Front Psychiatry. 2023 Mar 23;14:1145606. doi: 10.3389/fpsyt.2023.1145606 (PMC10076651; doi:10.3389/fpsyt.2023.1145606)
Supplement: Supplementary file 1 [file Data_Sheet_1.docx]

**Supplementary Material**

**Association of burnout and depression of pharmacists：A Network Analysis**

1. Table S1. Spearman correlation of the of the Emotional exhaustion, Depersonalization, Reduce professional efficacy and Depression
2. Figure S1. Accuracy of edge weights of BD network
3. Figure S2. Bootstrapped difference test for edge weights of BD network
4. Figure S3. Stability of node expected influences of BD network
5. Figure S4. Bootstrapped difference test for node expected influences of BD network
6. Figure S5. Stability of node bridge expected influences of BD network
7. Figure S6. Bootstrapped difference test for node bridge expected influences of BD network
8. Figure S7. Network comparisons of BD by genders, age, education level, professional title and length of service in pharmacists

**Table S1**

Spearman correlation of the of the Emotional exhaustion, Depersonalization, Reduce professional efficacy and Depression

|  | Emotional exhaustion | Depersonalization | Reduce professional efficacy | Depression |
| --- | --- | --- | --- | --- |
| Emotional exhaustion | 1 |  |  |  |
| Depersonalization | 0.652** | 1 |  |  |
| Reduce professional efficacy | 0.118** | 0.326** | 1 |  |
| Depression | 0.520** | 0.546** | 0.408** | 1 |

** : p < 0.01


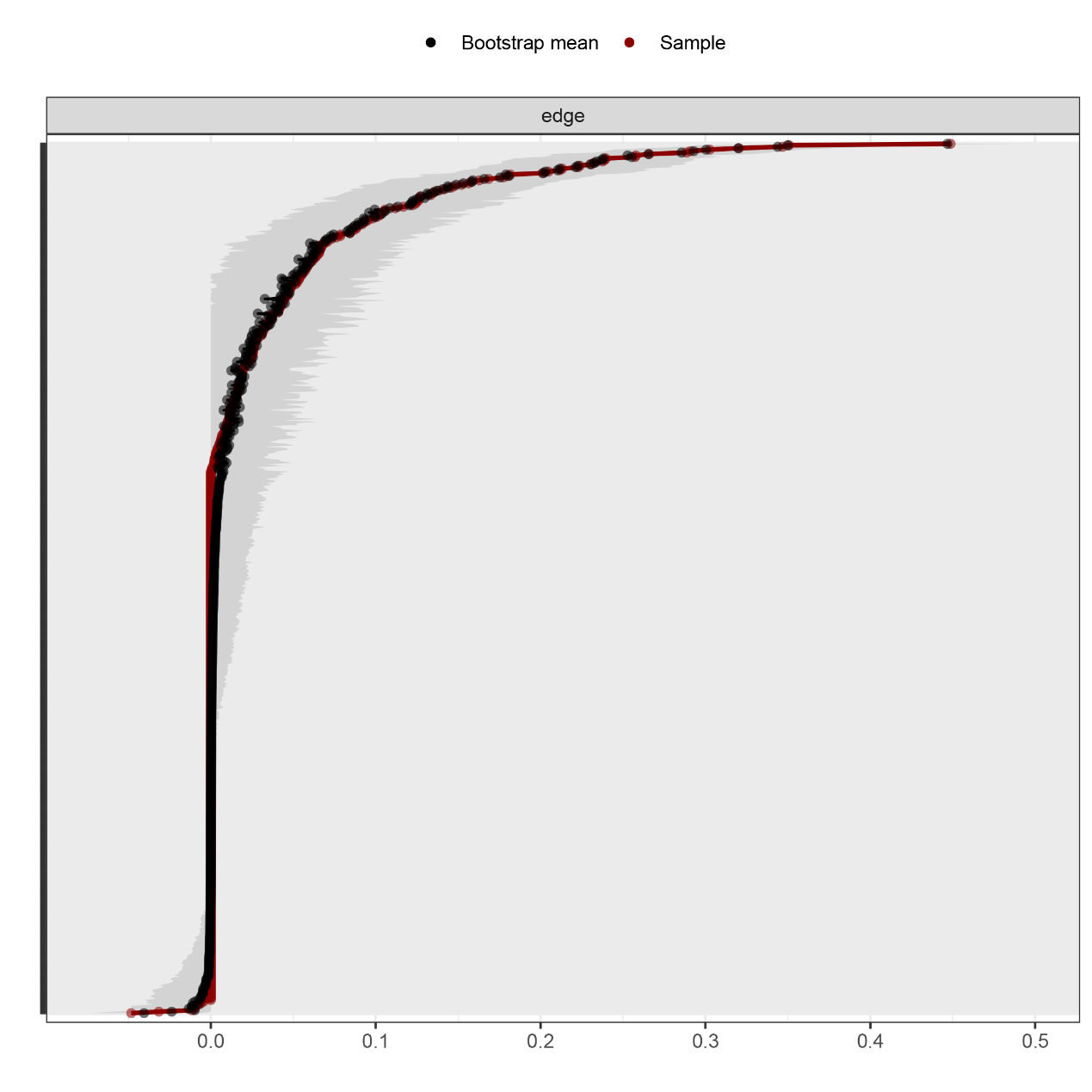
Figure S1. Accuracy of edge weights of BD network

*Note*: The red line depicts the sample edge weights and the gray bar depicts the bootstrapped confidence interval.


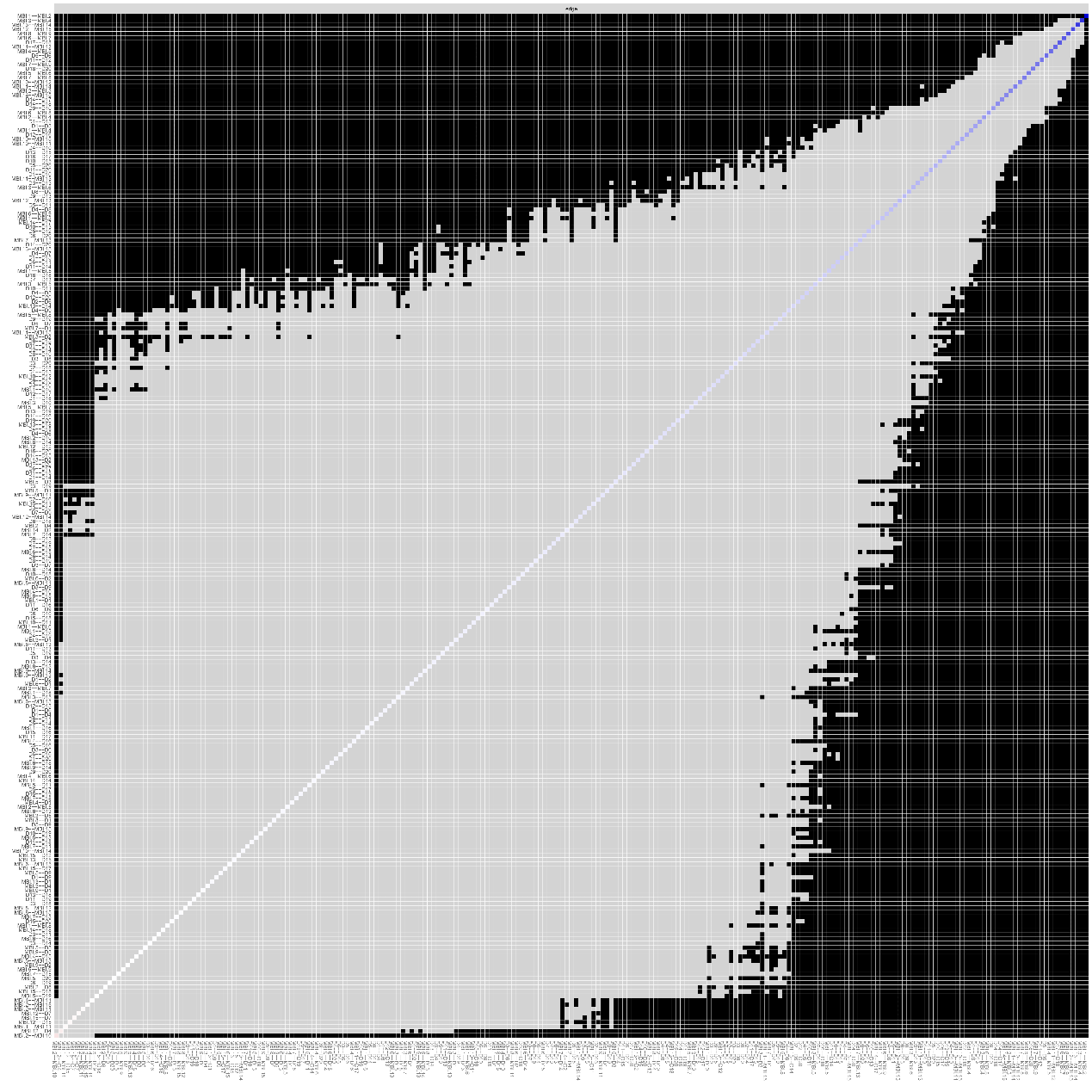


Figure S2. Bootstrapped difference test for edge weights of BD network

*Note*: Gray boxes indicate edge weights that do not differ significantly from one another, while black boxes indicate edge weights that do differ significantly. Blue and red boxes on the diagonal correspond to edge weights with positive and negative correlations, respectively.


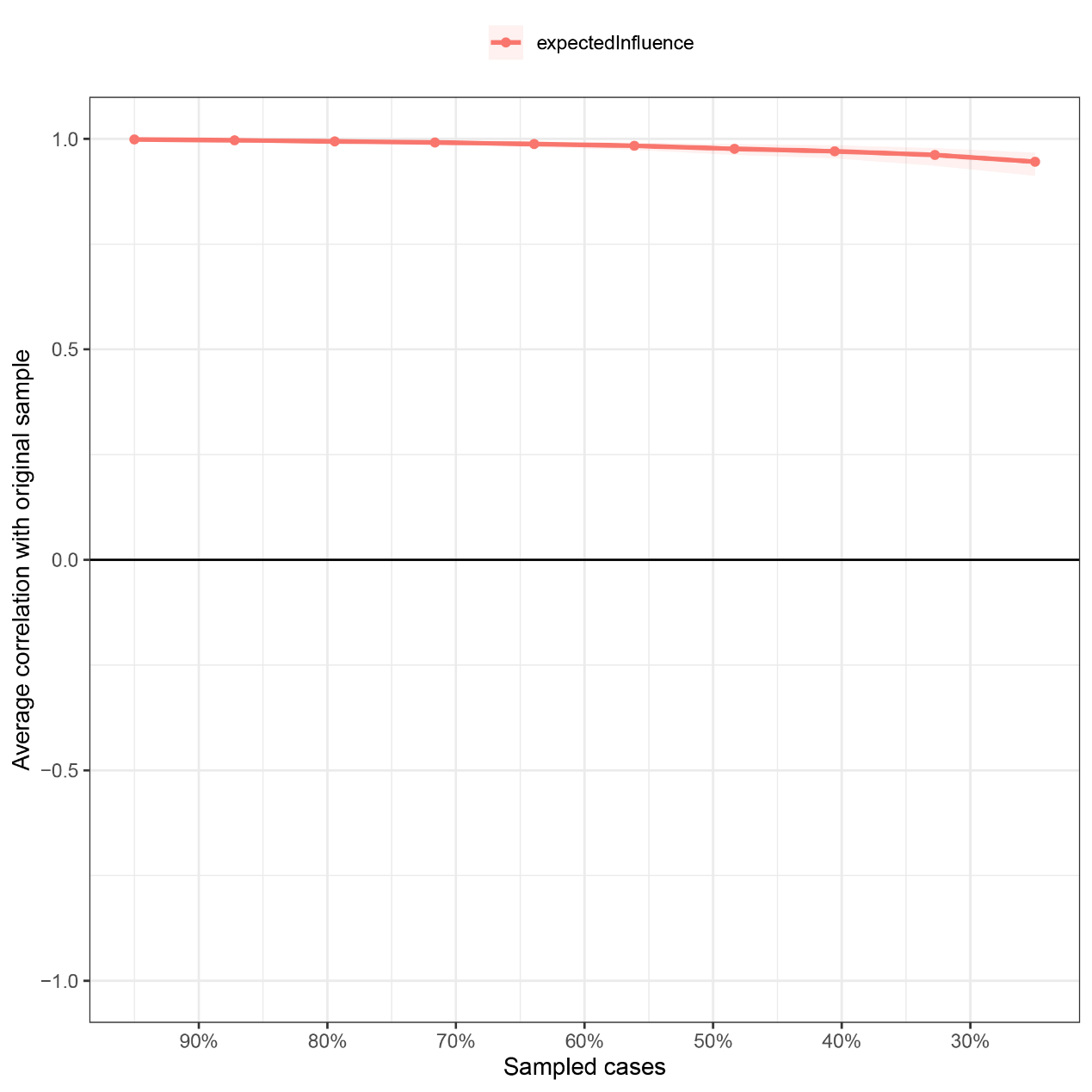


Figure S3. Stability of node expected influences of BD network

*Note*: The red line represents the average correlation between node expected influences in the full sample and subsample with the red area depicting the 2.5th quantile to the 97.5th quantile.


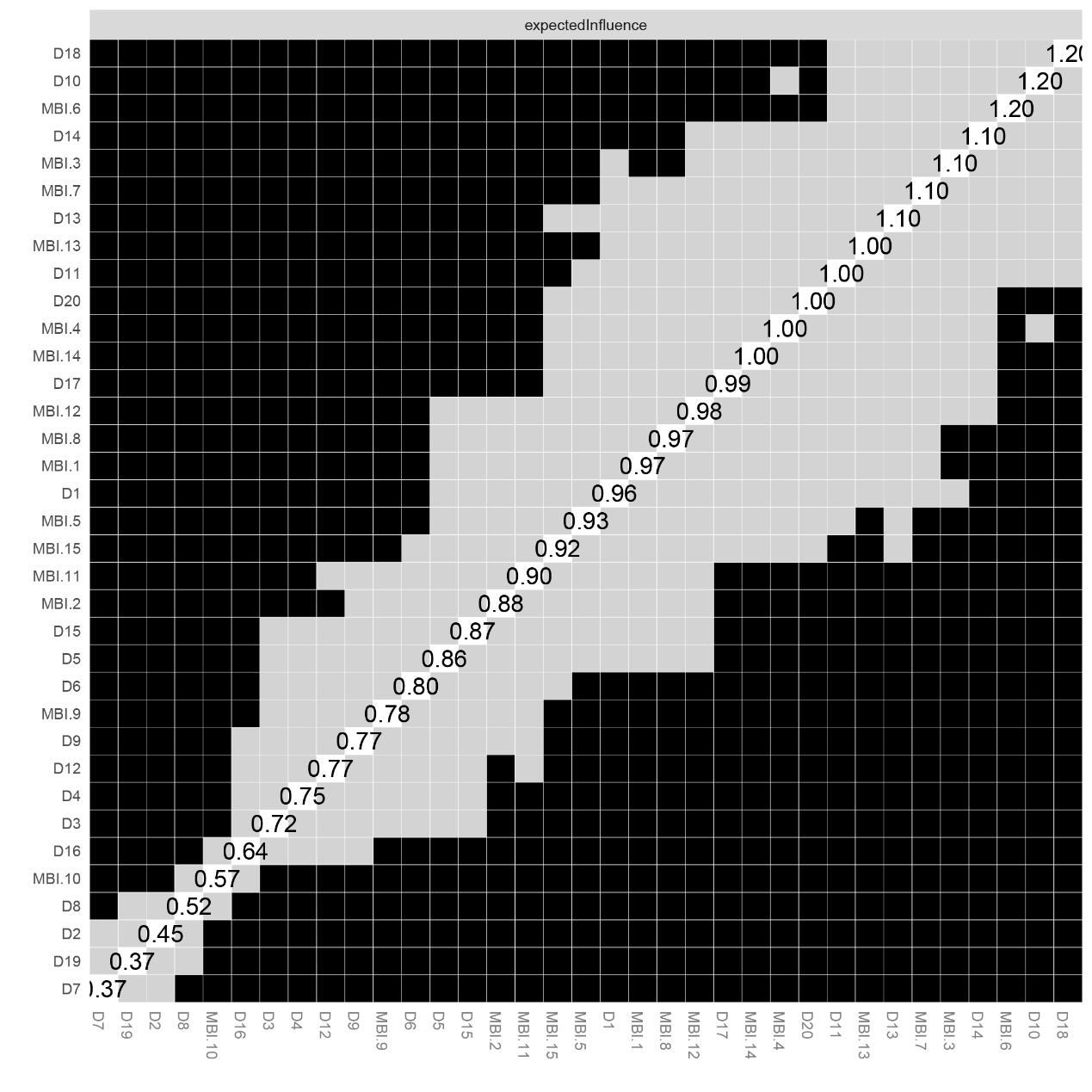


Figure S4. Bootstrapped difference test for node expected influences of BD network

*Note*: Gray boxes indicate node expected influences that do not differ significantly from one another, while black boxes indicate node expected influences that do differ significantly.


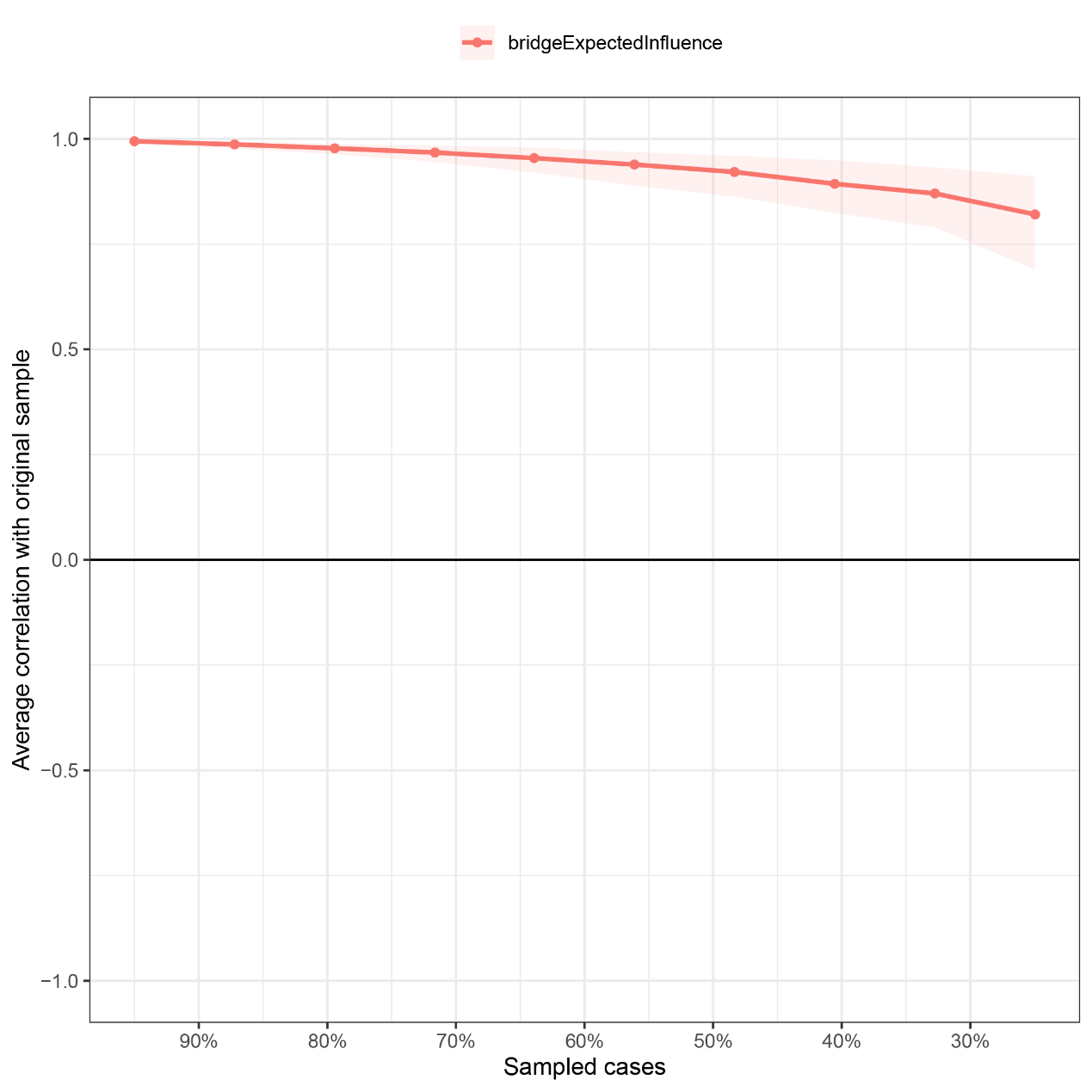


Figure S5. Stability of node bridge expected influences of BD network

*Note*: The red line represents the average correlation between node bridge expected influences in the full sample and subsample with the red area depicting the 2.5th quantile to the 97.5th quantile.


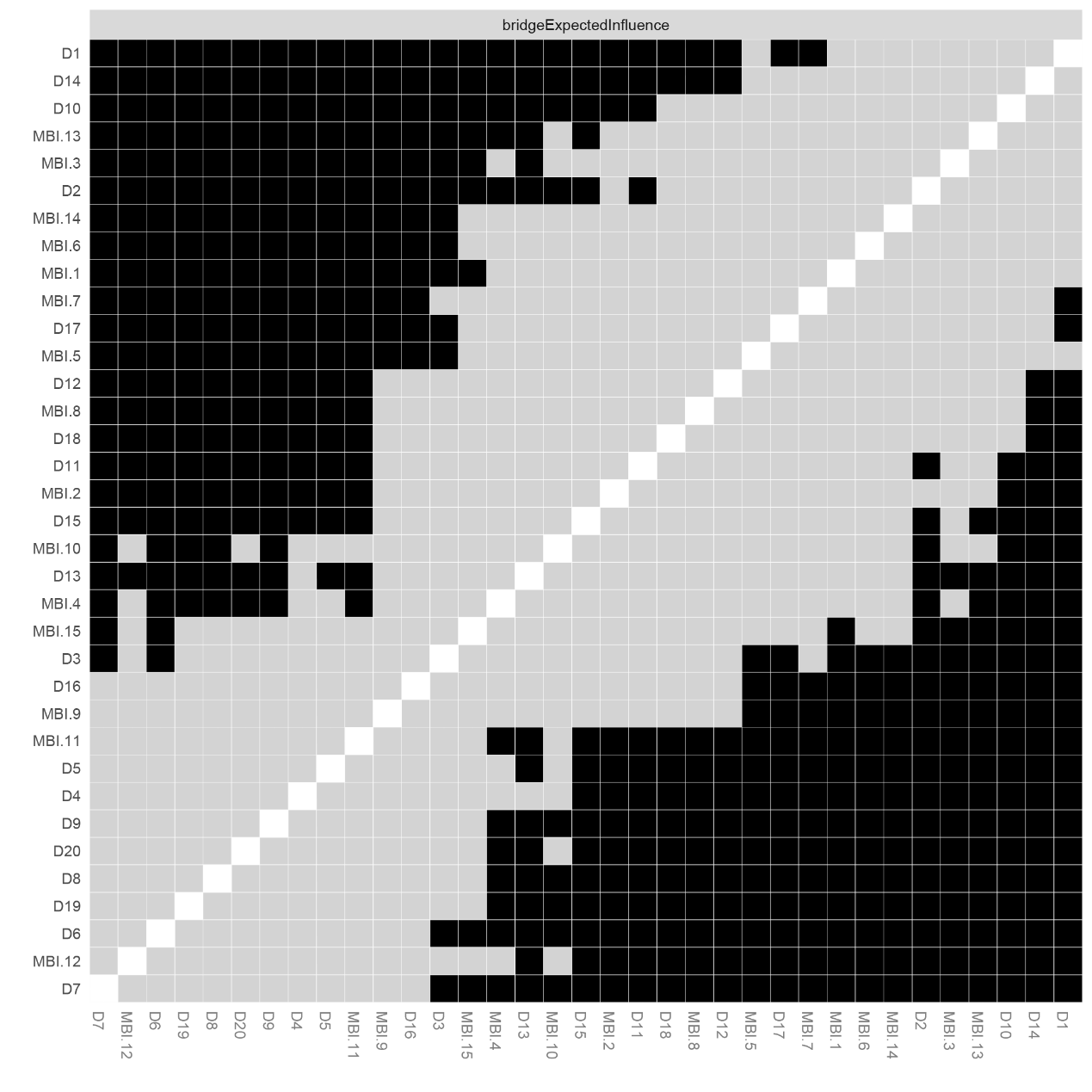


Figure S6. Bootstrapped difference test for node bridge expected influences of BD network

*Note*: Gray boxes indicate node bridge expected influences that do not differ significantly from one another, while black boxes indicate node bridge expected influences that do differ significantly.


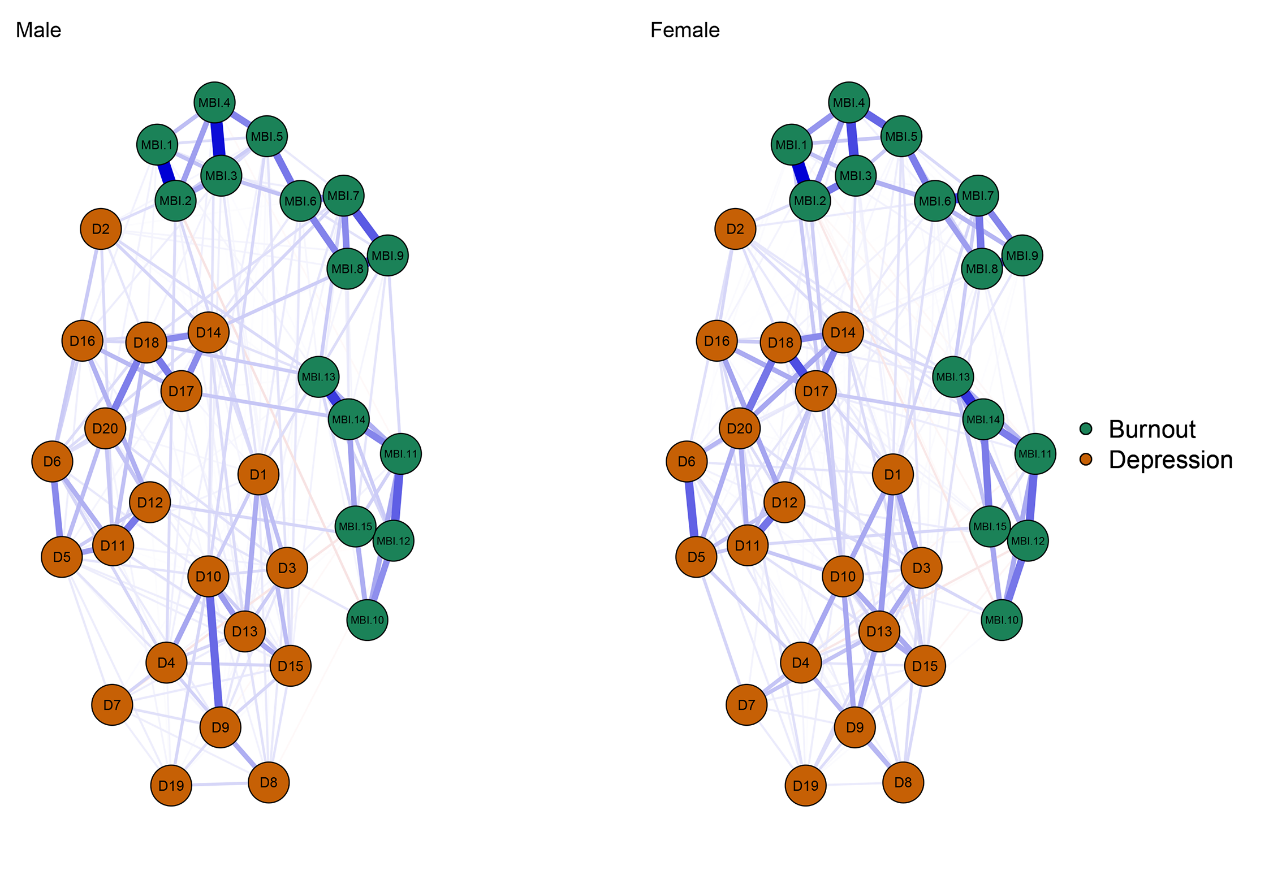


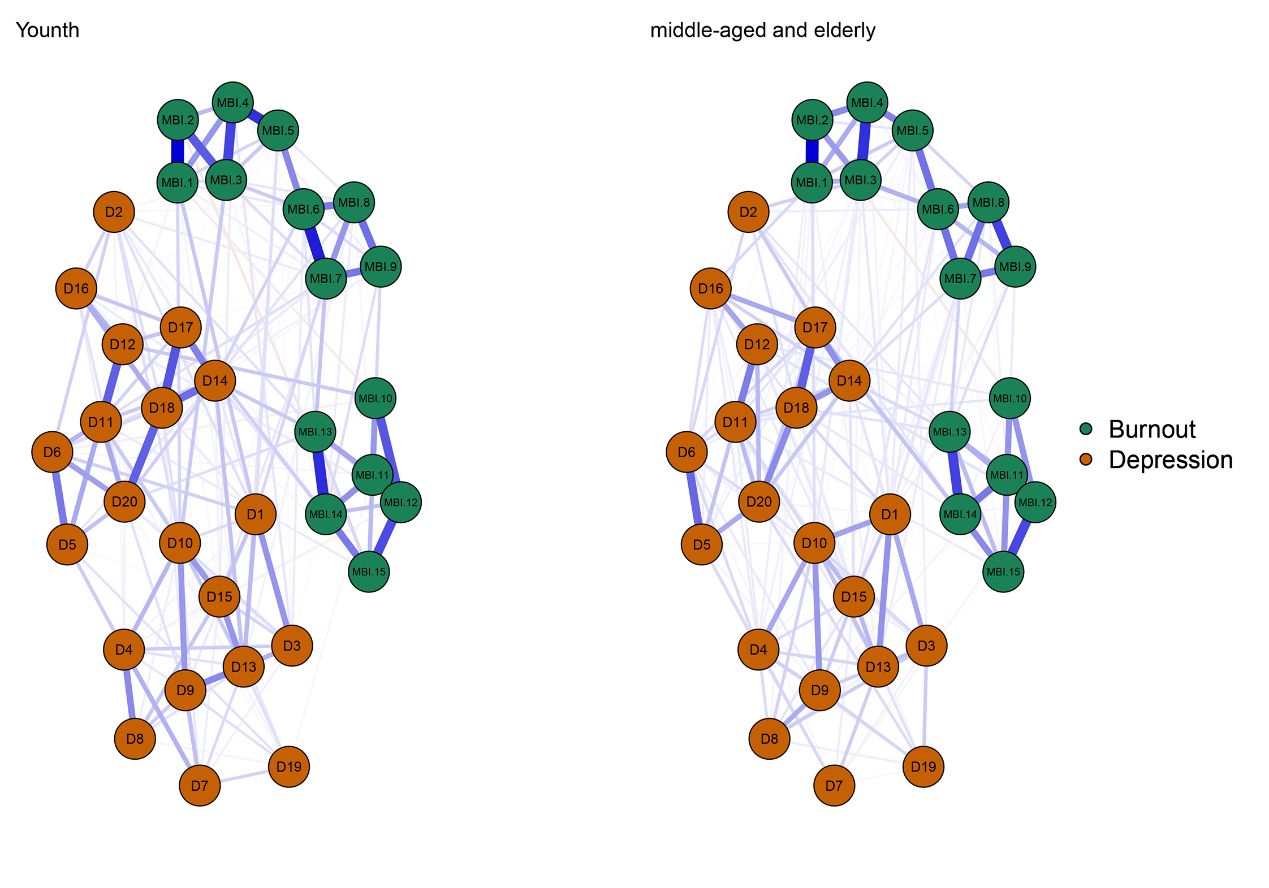


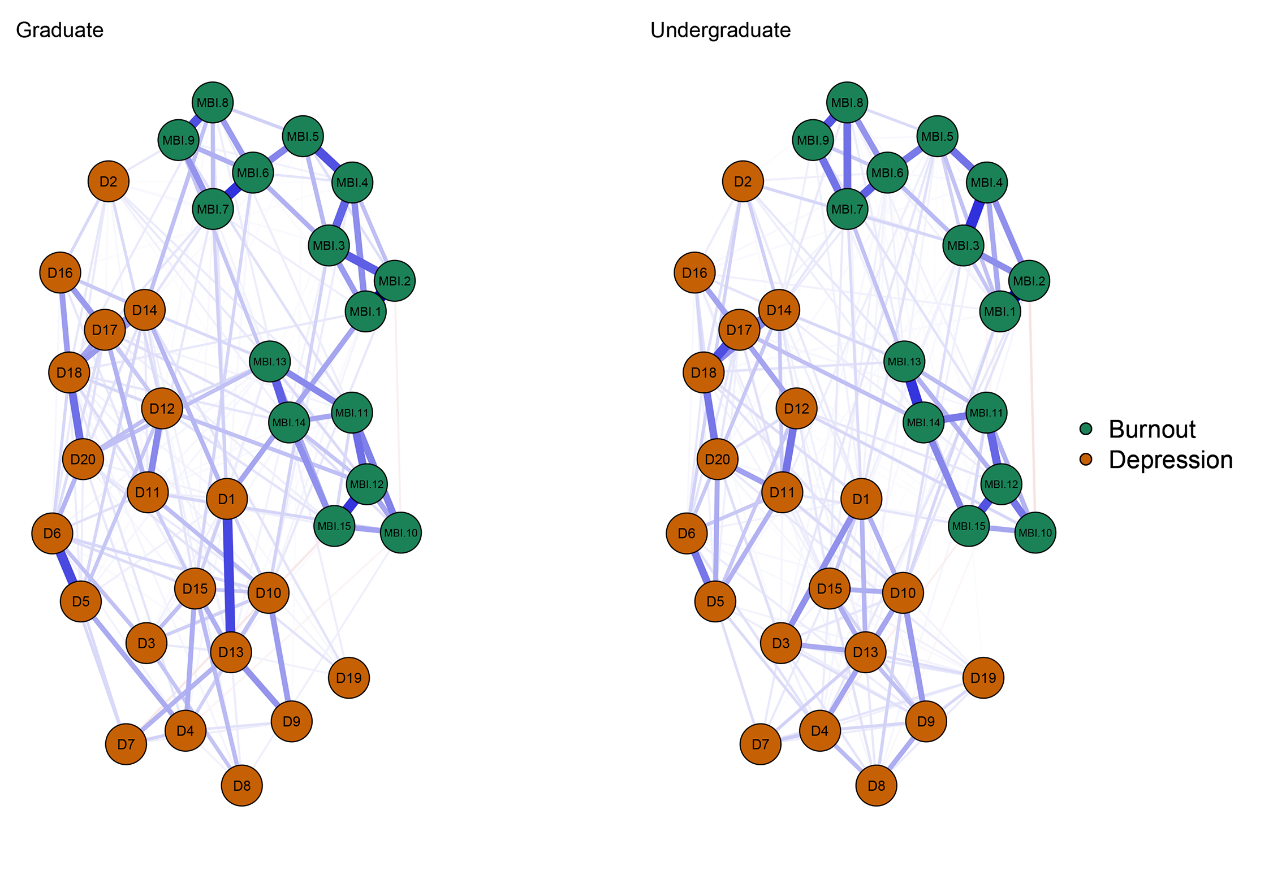

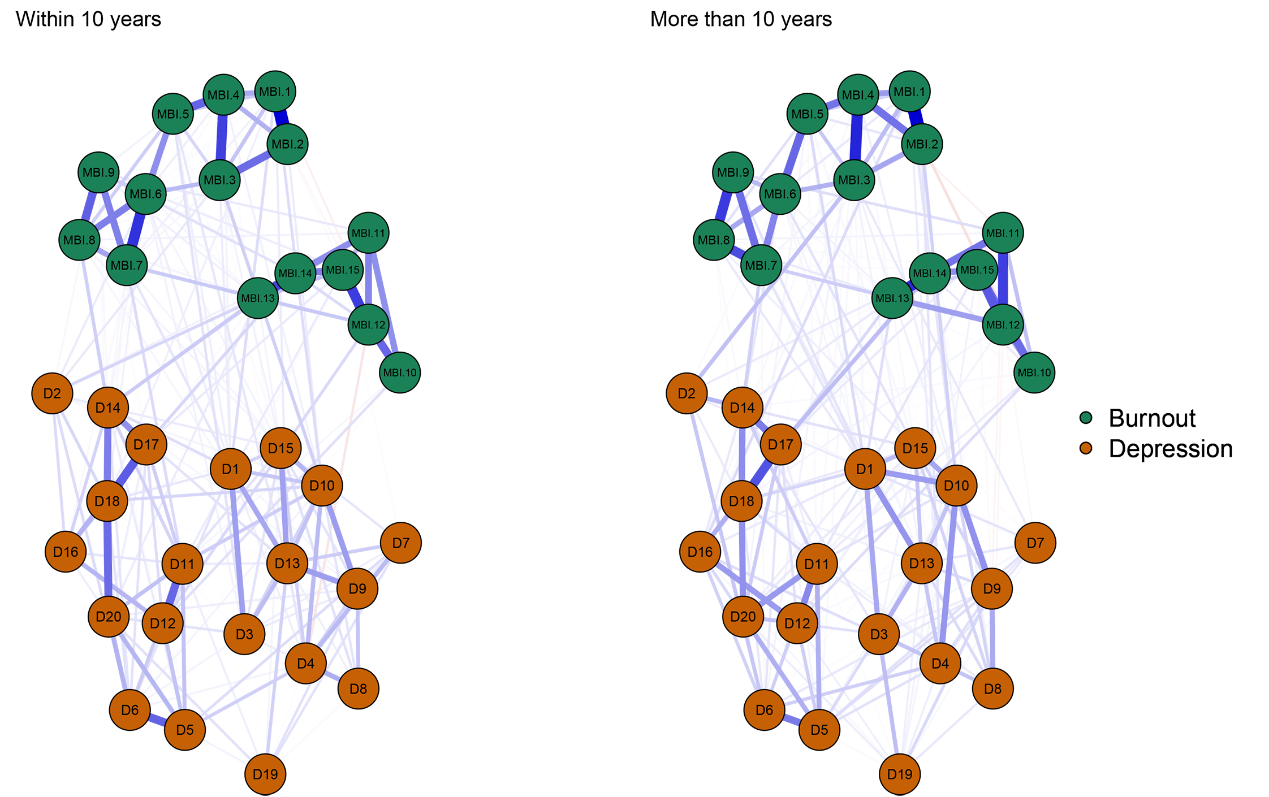


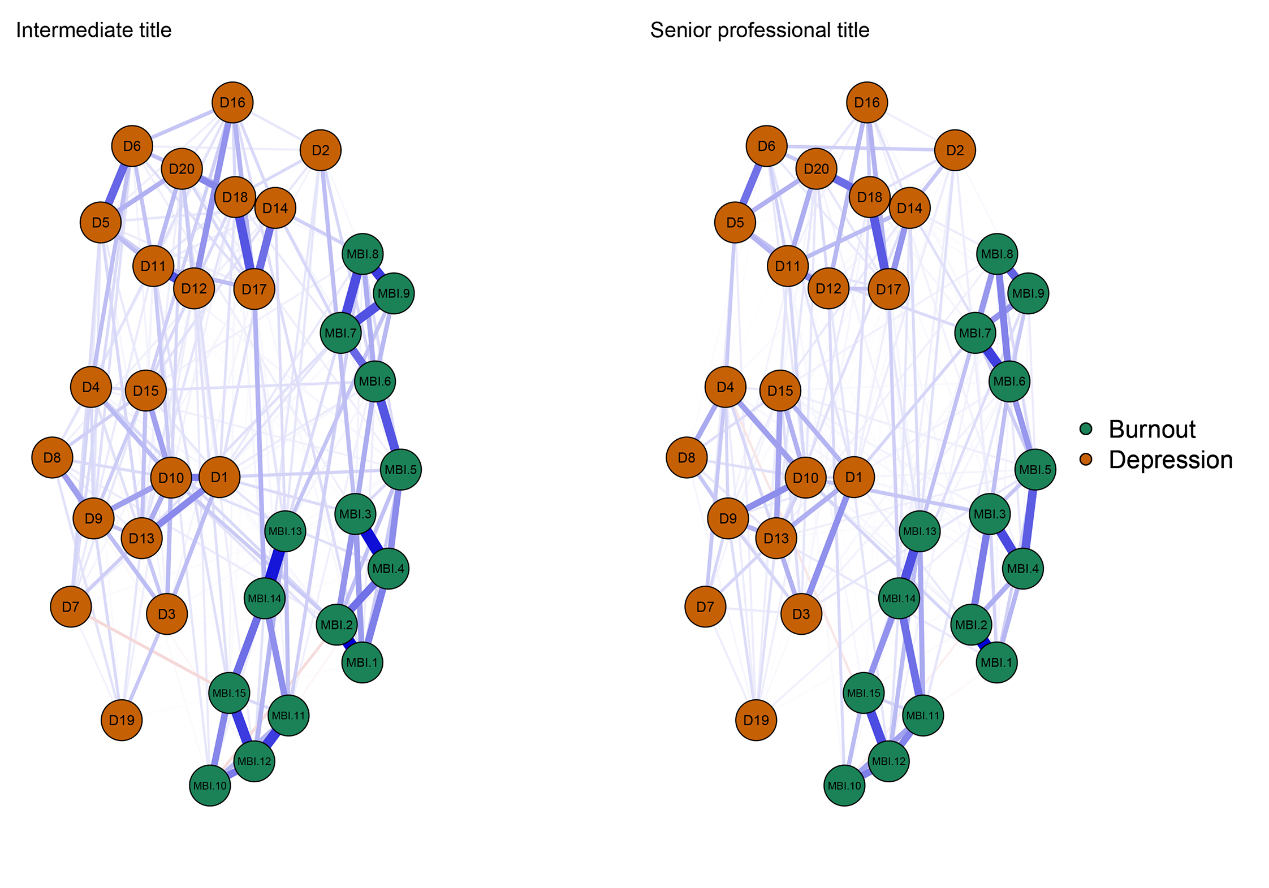


Figure S7. Network comparisons of BD by genders, age, education level, professional title and length of service in pharmacists
